# SSRT-DETR: Domain-Adaptive Semi-Supervised Detector

**DOI:** 10.3390/s26051539

**Published:** 2026-02-28

**Authors:** Wenshuai Zhang, Dong Zhou, Wenjie Xie, Wenrui Wang

**Affiliations:** The Research Institute of Electronic Science and Technology, University of Electronic Science and Technology of China, Chengdu 610054, China

**Keywords:** domain-adaptive object detection, RT-DETR, semi-supervised learning

## Abstract

Domain-adaptive object detection under set-prediction paradigms remains challenging, as Hungarian matching is sensitive to domain shift and fixed pseudo-label thresholds cannot simultaneously handle class imbalance and scene variability. This paper presents SSRT-DETR, a semi-supervised, domain-adaptive framework built on the real-time detector RT-DETR. We adopt a mean teacher–student architecture with style-transferred images to jointly model source and target domains. To stabilize the assignment process during the early stages of cross-domain training, Domain-Aware Matching (DAM) is formulated to augment the Hungarian matching cost with a teacher-guided decoder-query consistency term. Leveraging the more stable EMA teacher representations, DAM guides early matching toward domain-consistent assignments and is gradually annealed to recover standard matching as training converges. In parallel, we introduce Class-/Scene-Adaptive Pseudo-Labeling (CAP) to address a key limitation of existing DAOD methods that rely on fixed or globally tuned pseudo-label thresholds, which struggle with class imbalance and scene-dependent difficulty under domain shift. CAP leverages per-class confidence statistics and multi-view consistency to adapt classification and IoU thresholds across classes and scenes, while temperature scaling and quality-weighted losses provide soft control over pseudo-label reliability. Experiments on standard benchmarks demonstrate the robustness of SSRT-DETR. On Cityscapes→Foggy Cityscapes, SSRT-DETR improves mAP@0.5 from 51.0 to 54.3. On KITTI→Cityscapes and Sim10K→Cityscapes, it achieves 67.3 AP and 64.9 AP on the car category, respectively, clearly outperforming the RT-DETR baseline while maintaining real-time efficiency. Notably, consistent gains are observed in rare categories and adverse weather scenarios, validating the effectiveness of the proposed DAM and CAP modules.

## 1. Introduction

In many real-world applications, such as autonomous driving, intelligent surveillance, and human–computer interaction, object detectors [1,2,3] are required to operate under conditions that differ substantially from those seen during training. Weather, illumination, sensor characteristics, and scene style can all change dramatically. This mismatch between training and test distributions, commonly referred to as domain shift [4,5,6], often leads to a significant drop in detection accuracy and has become a key bottleneck for large-scale deployment. Although modern deep learning-based detectors can achieve human-level or even super-human performance on standard benchmarks, their accuracy tends to degrade noticeably once they are transferred to new cities, seasons, or imaging conditions.

To address this issue, domain-adaptive object detection (DAOD) [7,8,9] aims to exploit labeled source-domain data and unlabeled target-domain data to reduce the feature distribution gap between domains and improve target-domain performance. Existing approaches can be roughly grouped into three families: methods that explicitly minimize statistical measures of distribution discrepancy, reconstruction- or generation-based methods that capture shared structure, and adversarial learning-based methods that perform alignment at the image and instance levels. In particular, frameworks built on adversarial feature alignment and pseudo-label self-training have produced promising results for CNN-based two-stage detectors. However, most of these methods are tailored for architectures with region proposal networks (RPNs) [1,10], and their applicability and effectiveness for the rapidly emerging Transformer-based set-prediction detectors, such as DETR and RT-DETR [11], remain less explored.

Unlike traditional proposal-based detectors, DETR and its variants [12,13] perform one-to-one set prediction via Hungarian matching, thereby avoiding anchor design and post-processing heuristics. While this paradigm is conceptually elegant, it introduces new challenges in cross-domain scenarios. On the one hand, under severe domain shift, classification scores and bounding-box regression are highly unstable in the early stages of training, and a matching cost that relies solely on these signals is prone to incorrect assignments, which can amplify pseudo-label noise. On the other hand, existing pseudo-label-based semi-supervised or self-training methods typically use fixed confidence and IoU thresholds, which cannot simultaneously accommodate long-tailed categories, small objects, and complex scenes such as foggy or nighttime conditions, leading to ongoing debates on how to choose appropriate thresholds. These issues become particularly critical for the real-time detector RT-DETR [11], whose design emphasizes efficiency and simplicity, leaving limited room for heavy domain adaptation components.

To tackle these challenges, this paper proposes SSRT-DETR, a semi-supervised domain-adaptive framework built upon RT-DETR. The central idea is to enhance cross-domain robustness from two complementary perspectives—matching stability and pseudo-label quality control—while preserving real-time inference. Concretely, we introduce Domain-Aware Matching (DAM), which augments the Hungarian matching cost with a training-annealed domain discrepancy term driven by teacher–student decoder query similarity, thereby mitigating incorrect assignments at the early stages of cross-domain training. In parallel, we design Class-/Scene-Adaptive Pseudo-Labeling (CAP), which leverages per-class confidence distributions, multi-view consistency, temperature scaling, and quality-weighted supervision to perform fine-grained pseudo-label filtering and soft weighting, thus improving the effectiveness of supervision for long-tailed categories and adverse scenarios.

The present study aims to address two key questions: how to design a matching mechanism that is more robust to domain shift under the set-prediction paradigm, and how to exploit noisy target-domain pseudo-labels effectively without incurring additional inference cost. Through extensive experiments on standard adaptation benchmarks such as Cityscapes→Foggy Cityscapes, KITTI→Cityscapes, and Sim10K→Cityspaces, we show that SSRT-DETR can consistently improve cross-domain detection performance without sacrificing the real-time efficiency of RT-DETR, with particularly pronounced gains on rare categories and adverse weather conditions. Overall, the contributions of this work lie in providing an efficient yet robust domain-adaptation scheme for real-time Transformer-based detectors and in offering a new perspective on the interplay between matching and pseudo-label design within set-prediction frameworks.

In summary, our contributions are as follows:

- SSRT-DETR is introduced as a semi-supervised domain-adaptive framework for set-prediction-based object detectors, effectively mitigating matching instability and pseudo-label noise under domain shift while preserving real-time performance.
- We introduce Domain-Aware Matching (DAM), a teacher-guided matching strategy that stabilizes early-stage Hungarian assignment through an annealed domain-consistency term.
- We present Class-/Scene-Adaptive Pseudo-Labeling (CAP), which overcomes the limitations of fixed pseudo-label thresholds by adapting classification and IoU constraints across classes and scenes, combined with quality-weighted supervision.

## 2. Related Work

### 2.1. Transformer-Based Object Detection

Transformer-based object detection [14] has attracted extensive interest since the introduction of DETR [12], which replaces proposal-based pipelines with an end-to-end set prediction paradigm. DETR uses Hungarian matching for one-to-one assignment, eliminating anchor design and non-maximum suppression. However, vanilla DETR suffers from slow convergence and strong dependence on large-scale data. Subsequent works have introduced various enhancements: Deformable DETR [13] incorporates deformable attention to focus on sparse spatial regions and accelerates training; DINO [15] improves query initialization and localization precision through hybrid query selection and a two-stage architecture; and the RT-DETR family [11] achieves real-time performance through efficient multi-scale encoders and lightweight query selection, establishing itself as a practical choice for Transformer-based detection. Nevertheless, applying Transformer detectors to cross-domain scenarios remains challenging, as their global attention mechanisms often exacerbate feature distribution shifts, necessitating dedicated domain-adaptation strategies to ensure robustness.

### 2.2. Domain-Adaptive Object Detection

Unsupervised domain-adaptive Object Detection (UDA-OD) aims to reduce the distribution discrepancy between source and target domains, thereby mitigating performance degradation caused by domain shift. Early efforts were based on Faster R-CNN [8,9,16,17], leveraging gradient reversal layers or adversarial training to perform image-level and instance-level alignment, with the DANN family playing a pivotal role [4,5,18,19]. Subsequent studies introduced more refined alignment schemes, such as strong-weak alignment, task-specific alignment, and multi-level alignment, improving robustness for both classification and localization. However, these methods rely heavily on region proposal networks and are primarily used with two-stage detectors. With the rise of DETR, Transformer-based DAOD has become an emerging trend. Several works attempt to reduce domain gaps by introducing domain queries, encoder feature alignment, or masked reconstruction, whereas others integrate adversarial alignment, instance-level matching, or deformable attention into DETR frameworks [20,21]. Nevertheless, existing approaches have not fully addressed the matching instability inherent to set prediction under domain shift, nor have they adequately modeled how pseudo-label noise propagates within Transformer detectors, leaving room for further advancements.

### 2.3. Semi-Supervised and Pseudo-Label Learning for Set-Prediction Detectors

In semi-supervised and pseudo-label learning, teacher–student frameworks have become increasingly popular due to their ability to exploit unlabeled target-domain data without manual annotation [22,23,24,25,26,27,28]. Mean Teacher (MT) [29] and its variants employ consistency regularization and EMA-updated teachers to generate stable pseudo-labels for the student model. Subsequent works incorporate image-level alignment, confidence calibration, and other techniques to enhance pseudo-label reliability [30,31,32]. However, applying pseudo-label learning directly to set-prediction detectors introduces two major challenges [32,33]: first, the label assignment in DETR-like models depends on Hungarian matching, and unstable early-stage predictions can propagate matching noise into pseudo-labels; second, traditional pseudo-label filtering with fixed thresholds struggles to accommodate long-tailed class distributions and diverse scene conditions, resulting in uneven-quality pseudo-labels liable to mislead training. Although recent studies explore structured pseudo-labels, multi-view consistency, or masked reconstruction for Transformer detectors, few have focused on the coupled issue of matching stability and pseudo-label selection inherent to set prediction under domain shift. This gap motivates our SSRT-DETR framework, which explicitly addresses these intertwined challenges.

In summary, existing works have made significant progress in Transformer-based detection, domain adaptation, and pseudo-label learning. However, their combination under the set-prediction paradigm remains under-explored, particularly regarding the coupled issues of Hungarian matching instability and pseudo-label noise propagation under domain shift. This work focuses on this specific scope and proposes SSRT-DETR to address these challenges in a unified framework.

## 3. Materials and Methods

In this section, we introduce SSRT-DETR, a semi-supervised domain-adaptive framework built upon the real-time detector RT-DETR. We first formalize the problem setup and notations, then describe the base RT-DETR loss, the proposed Domain-Aware Matching (DAM), the Class-/Scene-Adaptive Pseudo-Labeling (CAP), the multi-view quality scoring, and finally the overall training objective.

**Figure 1:**
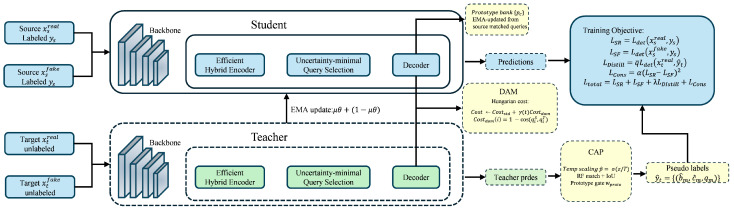
Overall pipeline of SSRT-DETR. A mean teacher–student architecture is trained with real/fake style-transferred views for both source and target domains. Domain-Aware Matching (DAM) augments Hungarian matching cost during training, while CAP generates quality-weighted pseudo-labels via Class-/Scene-adaptive thresholds, multi-view consistency, and prototype-based semantic verification.

As illustrated in Figure 1, SSRT-DETR follows a mean teacher–student training paradigm with real/fake style-transferred views. The teacher model generates pseudo-labels for target-domain images, while the student is trained using both labeled source data and quality-weighted target supervision. During training, Domain-Aware Matching (DAM) stabilizes Hungarian assignment in the early stages of cross-domain optimization, and Class-/Scene-Adaptive Pseudo-Labeling (CAP) filters and weights pseudo-labels to mitigate noise caused by class imbalance and scene variability. All components are jointly optimized in an end-to-end manner without introducing additional inference-time overhead.

### 3.1. Problem Setup and Notation

We consider the standard unsupervised domain adaptation setting for object detection, with a labeled source domain $D_{s}$ and an unlabeled target domain $D_{t}$. To reduce image-level domain discrepancy, both source and target samples are augmented with a style transfer network (e.g., CUT [34]), yielding “real/fake” dual views [35]. We denote

(1) $$D_{s}={\left\{\left(x_{s,n}^{\mathrm{real}},y_{s,n}\right),\left(x_{s,n}^{\mathrm{fake}},y_{s,n}\right)\right\}}_{n=1}^{N_{s}},$$

(2) $$D_{t}={\left\{x_{t,m}^{\mathrm{real}},x_{t,m}^{\mathrm{fake}}\right\}}_{m=1}^{N_{t}},$$

where each

(3) $$y_{s,n}={\left\{\left(b_{j},c_{j}\right)\right\}}_{j=1}^{N_{s,n}}$$

is a set of box-label pairs. The student detector is denoted by $F_{\theta }$, and the teacher by $F_{\theta^{\prime }}$, updated via EMA:

(4) $$\theta^{\prime }\leftarrow \mu \;\theta^{\prime }+\left(1-\mu \right)\;\theta ,\;\mu \in \left[0,1\right).$$

For each target image, the teacher produces a pseudo-label set

(5) $${\tilde{y}}_{t}={\left\{({\tilde{b}}_{m},{\tilde{c}}_{m},q_{m})\right\}}_{m=1}^{M}.$$

where ${\tilde{b}}_{m}$ is the predicted box, ${\tilde{c}}_{m}$ the class, and $q_{m}\in \left[0,1\right]$ the quality score. The native RT-DETR detection loss is written as $L_{\det}\left(x,y\right)$, comprising multi-level classification, L1 regression, GIoU, and denoising losses. On top of this base, SSRT-DETR introduces three key components: Domain-Aware Matching (DAM) to improve assignment robustness under domain shift, Class-/Scene-Adaptive Pseudo-Labeling (CAP) to enhance pseudo-label quality under long-tailed distributions and complex scenes, and multi-view quality scoring with temperature scaling to provide continuous weighting for pseudo-labels, thereby improving target-domain generalization without additional inference cost.

### 3.2. RT-DETR Base Detection Loss

In RT-DETR [36], the detection loss at each layer is determined by set prediction and Hungarian matching. Given an input image *x* and a target set

(6) $$y={\left\{\left(b_{j},c_{j}\right)\right\}}_{j=1}^{N},$$

the RT-DETR detection loss is as follows:

(7) $$L_{\det}\left(x,y\right)=\sum_{\ell =0}^{L}\left(\lambda_{\mathrm{cls}}L_{\mathrm{cls}}^{\left(\ell \right)}+\lambda_{\mathrm{bbox}}L_{\mathrm{bbox}}^{\left(\ell \right)}+\lambda_{\mathrm{giou}}L_{\mathrm{giou}}^{\left(\ell \right)}\right)-L_{\mathrm{dn}}.$$

For layer ℓ=0, it corresponds to the encoder head, and the remaining layers belong to the decoder; $\lambda_{\mathrm{cls}},\;\lambda_{\mathrm{bbox}},\;\lambda_{\mathrm{giou}}$ are scalar weights, and $L_{\mathrm{dn}}$ is the denoising loss. At layer *ℓ*, the Hungarian algorithm produces a matching set $M^{\left(\ell \right)}$, where $\left(i,j\right)\in M^{\left(\ell \right)}$ indicates that query *i* is matched to ground truth *j*. The classification loss is modeled with an IoU-weighted Focal/Varifocal Loss, yielding the following:

(8) $$y_{ij}\left(k\right)=\left\{\begin{array}{cc} s_{ij}, & k=c_{j},\\ 0, & k\neq c_{j}, \end{array}\right.$$

(9) $$L_{\mathrm{cls}}^{\left(\ell \right)}=\frac{1}{N_{q}}\sum_{i=1}^{N_{q}}\ell_{\mathrm{cls}}\left(z_{i}^{\left(\ell \right)},y_{i}^{\left(\ell \right)}\right),$$

where $N_{q}$ denotes the number of object queries in the decoder.

The L1 box loss and GIoU loss are defined as follows:

(10) $$L_{\mathrm{bbox}}^{\left(\ell \right)}=\frac{1}{|M^{\left(\ell \right)}|}\sum_{\left(i,j\right)\in M^{\left(\ell \right)}}{∥{\hat{b}}_{i}^{\left(\ell \right)}-b_{j}∥}_{1},$$

(11) $$L_{\mathrm{giou}}^{\left(\ell \right)}=\frac{1}{|M^{\left(\ell \right)}|}\sum_{\left(i,j\right)\in M^{\left(\ell \right)}}(1-\mathrm{GIoU}({\hat{b}}_{i}^{\left(\ell \right)},b_{j})).$$

Under severe domain shift and unstable early predictions, the matching set $M^{\left(\ell \right)}$ becomes a major source of error propagation, which motivates our domain-aware modification of the matching cost.

### 3.3. Domain-Aware Hungarian Matching (DAM)

The key challenge addressed by DAM is that Hungarian matching in set-prediction detectors relies entirely on student predictions, which are highly unreliable in the early stages of cross-domain training. This often leads to incorrect assignments that propagate errors to subsequent supervision. DAM alleviates this issue by introducing a teacher-guided regularization term into the matching cost, where the EMA-updated teacher provides more stable query representations to guide early assignments. Here, $\alpha_{\mathrm{cls}}$, $\alpha_{\mathrm{bbox}}$, and $\alpha_{\mathrm{giou}}$ denote the weighting coefficients for classification, box regression, and GIoU costs, respectively, and ρ controls the relative contribution of the domain-aware term.

**Figure 2:**
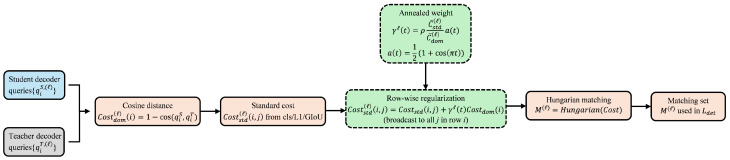
Domain-Aware Hungarian Matching (DAM). DAM augments the standard RT-DETR matching cost with a teacher–student query consistency term computed by cosine distance. The domain term is added as a row-wise regularizer with an annealed weight $\gamma^{\left(\ell \right)}\left(t\right)$, stabilizing early-stage assignments under domain shift without introducing inference-time overhead.

As shown in Figure 2, DAM augments the Hungarian matching cost with a teacher–student query consistency term, which stabilizes assignments under domain shift.

During early cross-domain training, student predictions on target-domain samples are highly unreliable, whereas vanilla Hungarian matching relies solely on these predictions to build the matching cost, making it vulnerable to incorrect assignments and amplifying subsequent supervision errors. To mitigate this, we introduce a domain-aware term that regularizes matching via consistency between teacher and student decoder queries. Let the student and teacher decoder queries at layer *ℓ* be

(12) $$q_{i}^{S,(\ell )},\;q_{i}^{T,(\ell )}\in R^{d}.$$

We define the domain cost via cosine distance:

(13) $${\mathrm{Cost}}_{\mathrm{dom}}^{\left(\ell \right)}\left(i\right)=1-\cos\left(q_{i}^{S,(\ell )},q_{i}^{T,(\ell )}\right)=1-\frac{\langle q_{i}^{S,(\ell )},q_{i}^{T,(\ell )}\rangle }{{∥q_{i}^{S,(\ell )}∥}_{2}{∥q_{i}^{T,(\ell )}∥}_{2}}.$$

Note that ${\mathrm{Cost}}_{\mathrm{dom}}^{\left(\ell \right)}\left(i\right)$ is a row-wise regularization term that depends only on the query index *i* and is broadcast to all candidate matches (i,j) during Hungarian assignment. The full matching cost at layer *ℓ* is then extended to

(14) $$\begin{array}{cc} {\mathrm{Cost}}^{\left(\ell \right)}\left(i,j\right)= & \;\alpha_{\mathrm{cls}}{\mathrm{Cost}}_{\mathrm{cls}}^{\left(\ell \right)}\left(i,j\right)+\alpha_{\mathrm{bbox}}{\mathrm{Cost}}_{\mathrm{bbox}}^{\left(\ell \right)}\left(i,j\right)\\ \; & +\alpha_{\mathrm{giou}}{\mathrm{Cost}}_{\mathrm{giou}}^{\left(\ell \right)}\left(i,j\right)+\gamma^{\left(\ell \right)}\left(t\right)\;{\mathrm{Cost}}_{\mathrm{dom}}^{\left(\ell \right)}\left(i\right). \end{array}$$

where the first three terms are derived from classification, L1, and GIoU losses, and $\gamma^{\left(\ell \right)}\left(t\right)$ is an annealed weight depending on the normalized training progress t∈[0,1]. To keep the domain term at a stable ratio ρ of the total cost, we estimate the average standard and domain costs within each batch,

(15) $${\bar{C}}_{\mathrm{std}}^{\left(\ell \right)}=\alpha_{\mathrm{cls}}E\left[{\mathrm{Cost}}_{\mathrm{cls}}^{\left(\ell \right)}\right]+\alpha_{\mathrm{bbox}}E\left[{\mathrm{Cost}}_{\mathrm{bbox}}^{\left(\ell \right)}\right]+\alpha_{\mathrm{giou}}E\left[{\mathrm{Cost}}_{\mathrm{giou}}^{\left(\ell \right)}\right],$$

(16) $${\bar{C}}_{\mathrm{dom}}^{\left(\ell \right)}=E\left[{\mathrm{Cost}}_{\mathrm{dom}}^{\left(\ell \right)}\right],$$

and set

(17) $$\gamma^{\left(\ell \right)}\left(t\right)=\rho \frac{{\bar{C}}_{\mathrm{std}}^{\left(\ell \right)}}{{\bar{C}}_{\mathrm{dom}}^{\left(\ell \right)}}\cdot a\left(t\right),$$

where ρ is a hyper-parameter controlling the relative contribution of the domain-aware cost term.

(18) $$a\left(t\right)=\frac{1}{2}\left(1+\cos(\pi t)\right),$$

where t∈[0,1] is the normalized training progress. Thus, the domain term dominates matching during early training and gradually vanishes, yielding domain-consistent assignments without affecting final convergence behavior.

DAM introduces a teacher-guided regularization term into Hungarian matching, which is gradually annealed to stabilize early assignments under domain shift.

### 3.4. Class-/Scene- and Prototype-Aware Pseudo-Labeling (CAP)

Existing pseudo-labeling strategies in domain-adaptive detection typically rely on fixed or globally tuned thresholds, which cannot simultaneously address class imbalance and scene-dependent difficulty under domain shift. As a result, pseudo-labels for rare classes or complex scenes are often either excessively noisy or overly suppressed. CAP is designed to address this limitation by adaptively adjusting confidence and IoU thresholds across classes and scenes, and by further integrating multi-view consistency and prototype-aware semantic verification. In single-class adaptation scenarios (e.g., KITTI→Cityscapes and Sim10K→Cityscapes), the class-adaptive components of CAP naturally degenerate to a class-agnostic form and therefore do not affect the training dynamics or inference behavior.

**Figure 3:**
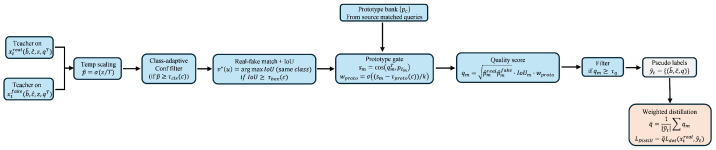
Class-/Scene- and Prototype-Aware Pseudo-Labeling (CAP). The EMA teacher predicts on-target real/fake views. After temperature scaling, candidates are filtered by class-adaptive confidence thresholds and paired across views via same-class IoU matching. A prototype-based semantic gate provides a soft weight $w_{\mathrm{proto}}$, and the final quality score $q_{m}=\sqrt{{\hat{p}}_{m}^{real}{\hat{p}}_{m}^{fake}}\cdot IoU_{m}\cdot w_{\mathrm{proto},m}$ is used for pseudo-label selection and quality-weighted distillation.

As illustrated in Figure 3, CAP generates pseudo-label candidates from the EMA teacher on both real/fake views, then filters and weights them using adaptive thresholds, multi-view consistency, and prototype-aware semantic verification.

Pseudo-label quality is the key factor that determines whether target-domain learning remains stable in semi-supervised domain-adaptive detection. While conventional CAP reduces noise via class-adaptive confidence filtering and real–fake geometric consistency, it may still retain class-confusion pseudo-labels that are confident and geometrically stable yet semantically incorrect under severe domain shift. To address this, we fully integrate a *prototype-aware* semantic consistency mechanism into CAP. The resulting CAP enforces pseudo-label reliability with three coupled constraints: (i) frequency-adaptive confidence filtering, (ii) frequency-adaptive geometric consistency, and (iii) prototype-based semantic consistency in feature space. All constraints are unified into a continuous quality score used for filtering and distillation weighting, without requiring any additional target annotations or introducing inference-time overhead.

#### Relative-Frequency Normalization

To avoid dataset-specific hard thresholds (e.g., $N_{c}>3000$), we define all class-adaptive thresholds as continuous functions of the *relative class frequency*. Let $N_{c}$ be the number of source samples for class *c*. We compute a (log-)normalized frequency

(19) $$N_{\min}=\min_{c}N_{c},\;N_{\max}=\max_{c}N_{c},\;r_{c}=\frac{\log\left(N_{c}+\epsilon \right)-\log\left(N_{\min}+\epsilon \right)}{\log\left(N_{\max}+\epsilon \right)-\log\left(N_{\min}+\epsilon \right)}\in \left[0,1\right],$$

where ϵ>0 is a numerical stabilizer. Intuitively, $r_{c}\approx 1$ indicates frequent classes and $r_{c}\approx 0$ indicates rare classes.

We define a class-specific confidence threshold as a monotone decreasing function of $r_{c}$, so that frequent classes use looser thresholds while rare classes use stricter ones:

(20) $$\tau_{\mathrm{cls}}\left(c\right)=\tau_{\mathrm{cls}}^{\max}-r_{c}\left(\tau_{\mathrm{cls}}^{\max}-\tau_{\mathrm{cls}}^{\min}\right).$$

Similarly, to impose stronger geometric stability for rare classes, we define the class-specific real–fake IoU threshold (also monotone decreasing in $r_{c}$):

(21) $$\tau_{\mathrm{box}}\left(c\right)=\tau_{\mathrm{box}}^{\max}-r_{c}\left(\tau_{\mathrm{box}}^{\max}-\tau_{\mathrm{box}}^{\min}\right).$$

For each target image, the EMA teacher predicts on both $x_{t}^{\mathrm{real}}$ and $x_{t}^{\mathrm{fake}}$. For view v∈{real,fake}, we denote the candidate set as

(22) $$B^{v}={\left\{\left({\hat{b}}_{k}^{v},{\hat{c}}_{k}^{v},{\hat{p}}_{k}^{v},q_{k}^{T,(\ell ),v}\right)\right\}}_{k},$$

where ${\hat{b}}_{k}^{v}$ is the box, ${\hat{c}}_{k}^{v}$ the predicted class, ${\hat{p}}_{k}^{v}$ the temperature-scaled confidence, and $q_{k}^{T,(\ell ),v}\in R^{d}$ the decoder query feature at layer *ℓ*. We first apply class-adaptive confidence filtering by retaining candidates satisfying

(23) $${\hat{p}}_{k}^{v}\geq \tau_{\mathrm{cls}}\left({\hat{c}}_{k}^{v}\right).$$

Then, for each remaining fake-view candidate $\left({\hat{b}}_{u}^{\mathrm{fake}},{\hat{c}}_{u}^{\mathrm{fake}}\right)$, we find the best same-class match in the real view:

(24) $$v^{*}\left(u\right)=\arg\max_{v:{\hat{c}}_{v}^{\mathrm{real}}={\hat{c}}_{u}^{\mathrm{fake}}}\mathrm{IoU}\left({\hat{b}}_{u}^{\mathrm{fake}},{\hat{b}}_{v}^{\mathrm{real}}\right),$$

and keep the pair only if the class-adaptive IoU constraint holds:

(25) $${\mathrm{IoU}}_{m}:=\mathrm{IoU}\left({\hat{b}}_{u}^{\mathrm{fake}},{\hat{b}}_{v^{*}\left(u\right)}^{\mathrm{real}}\right)\geq \tau_{\mathrm{box}}\left({\hat{c}}_{u}^{\mathrm{fake}}\right).$$

We set the pseudo-label class as ${\tilde{c}}_{m}={\hat{c}}_{u}^{\mathrm{fake}}$ and use the real-view box ${\tilde{b}}_{m}={\hat{b}}_{v^{*}\left(u\right)}^{\mathrm{real}}$ as supervision since the student is trained on $x_{t}^{\mathrm{real}}$.

Confidence and IoU constraints alone may still retain semantically wrong yet stable pseudo-labels. We therefore introduce a prototype-aware semantic consistency term. On the labeled source branch, we maintain one prototype vector per class using *high-confidence* matched decoder queries. Let $M^{\left(\ell \right)}$ denote the Hungarian matching set at layer *ℓ*. For $\left(i,j\right)\in M^{\left(\ell \right)}$, query *i* is matched to ground-truth *j* of class $c_{j}$, with student query feature $q_{i}^{S,(\ell )}\in R^{d}$. We update prototypes only when the student confidence on the GT class satisfies $p_{i,c_{j}}^{S,(\ell )}\geq \tau_{\mathrm{src}}$. Define

(26) $$I_{c}^{\left(\ell \right)}=\left\{i|\left(i,j\right)\in M^{\left(\ell \right)},\;c_{j}=c,\;p_{i,c}^{S,(\ell )}\geq \tau_{\mathrm{src}}\right\},$$

where $\tau_{\mathrm{src}}$ is a confidence threshold used to select reliable source-domain queries for prototype updating. And compute the batch mean of normalized queries:

(27) $${\bar{q}}_{c}^{\left(\ell \right)}=\frac{1}{|I_{c}^{\left(\ell \right)}|}\sum_{i\in I_{c}^{\left(\ell \right)}}\frac{q_{i}^{S,(\ell )}}{∥q_{i}^{S,(\ell )}{∥}_{2}}.$$

We then update the class prototype with EMA and re-normalize

(28) $$p_{c}\leftarrow \left(1-\beta \right)p_{c}+\beta \;{\bar{q}}_{c}^{\left(\ell \right)},\;p_{c}\leftarrow \frac{p_{c}}{∥p_{c}{∥}_{2}},$$

where β∈(0,1] is the prototype update rate.

For each target pseudo-label candidate that passes the confidence and IoU constraints, we compute its query–prototype cosine similarity. We represent the candidate by averaging normalized teacher queries from the real and fake views:

(29) $$q_{m}^{T,(\ell )}=\frac{1}{2}\left(\frac{q_{u}^{T,(\ell ),\mathrm{fake}}}{∥q_{u}^{T,(\ell ),\mathrm{fake}}{∥}_{2}}+\frac{q_{v^{*}\left(u\right)}^{T,(\ell ),\mathrm{real}}}{∥q_{v^{*}\left(u\right)}^{T,(\ell ),\mathrm{real}}{∥}_{2}}\right),$$

and compute

(30) $$s_{m}=\cos\left(q_{m}^{T,(\ell )},p_{{\tilde{c}}_{m}}\right)=\frac{\langle q_{m}^{T,(\ell )},p_{{\tilde{c}}_{m}}\rangle }{{∥q_{m}^{T,(\ell )}∥}_{2}\;{∥p_{{\tilde{c}}_{m}}∥}_{2}}.$$

To enforce stricter prototype constraints for frequent classes while being more tolerant for rare classes, we define a frequency-adaptive prototype threshold as a monotone increasing function of $r_{c}$:

(31) $$\tau_{\mathrm{proto}}\left(c\right)=\tau_{\mathrm{proto}}^{\min}+r_{c}\left(\tau_{\mathrm{proto}}^{\max}-\tau_{\mathrm{proto}}^{\min}\right).$$

We then obtain a soft prototype weight by a sigmoid mapping:

(32) $$w_{m}^{\mathrm{proto}}=\sigma \;\left(\frac{s_{m}-\tau_{\mathrm{proto}}\left({\tilde{c}}_{m}\right)}{\kappa }\right),$$

where κ>0 controls the softness of the gate.

We apply temperature scaling to teacher logits *z* to obtain calibrated confidences,

(33) $$\hat{p}=\sigma \left(z/T\right),$$

and define the multi-view base quality using the geometric mean:

(34) $$q_{m}^{\mathrm{mv}}=\sqrt{{\hat{p}}_{m}^{\mathrm{real}}{\hat{p}}_{m}^{\mathrm{fake}}}\cdot {\mathrm{IoU}}_{m}.$$

Finally, CAP integrates prototype semantics into the quality score:

(35) $$q_{m}=q_{m}^{\mathrm{mv}}\cdot w_{m}^{\mathrm{proto}}=\sqrt{{\hat{p}}_{m}^{\mathrm{real}}{\hat{p}}_{m}^{\mathrm{fake}}}\cdot {\mathrm{IoU}}_{m}\cdot \sigma \;\left(\frac{s_{m}-\tau_{\mathrm{proto}}\left({\tilde{c}}_{m}\right)}{\kappa }\right).$$

We retain pseudo-labels with $q_{m}\geq \tau_{q}$ to form ${\tilde{y}}_{t}={\left\{\left({\tilde{b}}_{m},{\tilde{c}}_{m},q_{m}\right)\right\}}_{m=1}^{M}$, and use $q_{m}$ (or its batch average) to weight the distillation loss. By unifying confidence, geometry, and prototype-based semantic consistency, CAP effectively reduces class-confusion noise and improves pseudo-label reliability and balance, particularly for rare categories.

### 3.5. Pseudo-Label Quality Weighting with Temperature Scaling

After CAP filtering, pseudo-labels still present a continuous spectrum of reliability. Instead of using a hard accept/reject decision only, we leverage the CAP quality score to softly weight the contribution of pseudo-label supervision.

Let the teacher’s logits for the *m*-th candidate in the fake/real views be $z_{m}^{fake}$ and $z_{m}^{real}$. We obtain calibrated confidences via temperature scaling:

(36) $${\hat{p}}_{m}^{fake}=\sigma \;\left(z_{m}^{fake}/T\right),\;{\hat{p}}_{m}^{real}=\sigma \;\left(z_{m}^{real}/T\right),$$

where T>0 is the temperature. Denote by $IoU_{m}$ the IoU of the matched real–fake boxes computed in CAP. Following the multi-view design in Section 3.4, we define the base multi-view quality as the geometric mean:

(37) $$q_{mv,m}=\sqrt{{\hat{p}}_{m}^{real}{\hat{p}}_{m}^{fake}}\cdot IoU_{m}.$$

To further suppress semantically inconsistent yet geometrically stable pseudo-labels under domain shift, we incorporate the prototype-aware weight $w_{proto,m}$ computed in CAP:

(38) $$q_{m}=q_{mv,m}\cdot w_{proto,m}.$$

We retain pseudo-labels with $q_{m}\geq \tau_{q}$ to form ${\tilde{y}}_{t}={\left\{\left({\tilde{b}}_{m},{\tilde{c}}_{m},q_{m}\right)\right\}}_{m=1}^{M}$, where $\tau_{q}$ denotes the minimum quality threshold for pseudo-label selection.

For each target image, the average pseudo-label quality is computed as follows:

(39) $$\bar{q}=\frac{1}{|{\tilde{y}}_{t}|}\sum_{\left({\tilde{b}}_{m},{\tilde{c}}_{m},q_{m}\right)\in {\tilde{y}}_{t}}q_{m}.$$

The student is distilled on $x_{t}^{real}$ using pseudo-labels ${\tilde{y}}_{t}$ through

(40) $$L_{PL}=L_{det}\;\left(x_{t}^{real},{\tilde{y}}_{t}\right),\;L_{Distill}=\bar{q}\cdot L_{PL}.$$

When pseudo-label quality is high, $\bar{q}$ approaches 1 and target supervision becomes effective; when pseudo-labels are unreliable, $\bar{q}$ automatically down-weights the distillation signal, thereby suppressing noise in a continuous fashion.

### 3.6. Source Branches and Overall Objective

On the source domain, the student detector minimizes the RT-DETR detection loss on both real and stylized images,

(41) $$L_{\mathrm{SR}}=L_{\det}\left(x_{s}^{\mathrm{real}},y_{s}\right),$$

(42) $$L_{\mathrm{SF}}=L_{\det}\left(x_{s}^{\mathrm{fake}},y_{s}\right).$$

To encourage consistent detection performance under style changes, we introduce a source–fake consistency constraint:

(43) $$L_{\mathrm{Cons}}=\alpha {\left(L_{\mathrm{SR}}-L_{\mathrm{SF}}\right)}^{2}.$$

where α is a hyper-parameter. Combining source supervision, target-domain pseudo-label distillation, and consistency regularization, the overall loss of SSRT-DETR in each iteration is given by

(44) $$L_{\mathrm{total}}=L_{\mathrm{SR}}+L_{\mathrm{SF}}+\lambda L_{\mathrm{Distill}}+L_{\mathrm{Cons}},$$

(45) $$\begin{array}{cc} L_{\mathrm{total}}= & \;L_{\det}\left(x_{s}^{\mathrm{real}},y_{s}\right)+L_{\det}\left(x_{s}^{\mathrm{fake}},y_{s}\right)\\ \; & +\lambda \;\bar{q}\;L_{\det}\left(x_{t}^{\mathrm{real}},{\tilde{y}}_{t}\right)+\alpha {\left(L_{\det}\left(x_{s}^{\mathrm{real}},y_{s}\right)-L_{\det}\left(x_{s}^{\mathrm{fake}},y_{s}\right)\right)}^{2}. \end{array}$$

where λ balances supervised and unsupervised losses. Importantly, Domain-Aware Matching (DAM) modifies the internal matching cost of $L_{\det}$ and thus acts as a structural change to the detection loss rather than an additional explicit term, which implies no extra cost at inference time.

Compared with existing domain-adaptive object detection methods, SSRT-DETR introduces two key improvements specifically tailored for set-prediction detectors: (1) DAM stabilizes Hungarian matching during early cross-domain training, effectively reducing error propagation; (2) CAP improves pseudo-label reliability by jointly addressing class imbalance and scene variability through adaptive thresholding and quality-weighted supervision. These improvements are tightly integrated into the RT-DETR framework, leading to more stable optimization and better generalization under domain shifts.

## 4. Experiments

We conducted extensive experiments to validate the effectiveness of the proposed method on a variety of cross-domain adaptation benchmarks. These benchmarks cover diverse adaptation scenarios, including adverse weather adaptation (Cityscapes→Foggy Cityscapes), synthetic-to-real adaptation (Sim10K→Cityscapes), and cross-camera adaptation (KITTI→Cityscapes). In addition, comprehensive ablation studies were performed to analyze the contribution of each proposed module. Experimental results consistently demonstrate that our method is highly robust and adaptable across domain shifts, leading to notable improvements in generalization performance across multiple scene understanding tasks.

### 4.1. Datasets

**Cityscapes→Foggy Cityscapes.** Cityscapes [37] is a widely used urban scene understanding benchmark, consisting of 2975 training images and 500 validation images with high-quality pixel-level annotations. Foggy Cityscapes [38] is derived from Cityscapes by synthetically introducing fog effects with varying intensities. Specifically, three fog density levels (0.005, 0.01, and 0.02) are provided, corresponding to light, moderate, and heavy fog conditions, respectively. In our experiments, we adopt the most severe setting (fog level 0.02) to evaluate performance on the eight object categories shared by both domains.

**Sim10K→Cityscapes.** Sim10K [39] is a large-scale synthetic dataset generated using a game engine, containing 10,000 images annotated with object-level bounding boxes. In this adaptation scenario, Sim10K serves as the source domain, and Cityscapes as the target domain. Following standard practice, evaluation is conducted exclusively on the “car” category.

**KITTI→Cityscapes.** KITTI [3] is another real-world street scene dataset captured using different cameras and collected from cities distinct from those in Cityscapes, resulting in a noticeable domain gap. In this setting, we employ 7481 KITTI images for training and focus on the “car” category, which is the only class shared between the two datasets.

### 4.2. Implementation Details

To provide a clear overview of the proposed training pipeline, Algorithm 1 summarizes the complete optimization procedure of SSRT-DETR. Different from online style translation, the fake-view images used in our framework are generated *offline* using CUT and stored together with their corresponding real images. During training, each mini-batch therefore consists of paired real/fake samples from both source and target domains, enabling consistent dual-view learning without additional runtime cost.

The student model is optimized using supervised losses on the labeled source domain and quality-weighted pseudo-label supervision on the unlabeled target domain. The teacher model is updated by an exponential moving average (EMA) and is only used to generate pseudo-labels and guide matching. Notably, Domain-Aware Matching (DAM) is applied *inside* the Hungarian matching process when computing the detection loss, while Class-/Scene-Adaptive Pseudo-Labeling (CAP) is applied only on the target branch during pseudo-label generation and filtering.
**Algorithm 1:** Training Procedure of SSRT-DETR (offline fake images)
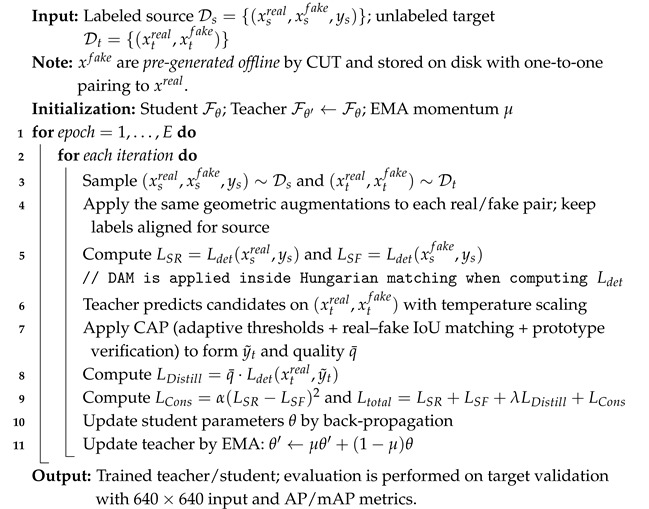


It is worth emphasizing that DAM and CAP play fundamentally different roles in the proposed framework. DAM modifies the internal assignment mechanism of set-prediction detectors by augmenting the Hungarian matching cost, and therefore affects how supervision signals are constructed during training. In contrast, CAP operates at the pseudo-label level and aims to control the reliability and contribution of unlabeled target supervision. This clear separation allows SSRT-DETR to jointly stabilize early-stage assignment and suppress noisy pseudo-label propagation, without introducing additional modules or inference-time overhead.

Table 1 reports all key hyperparameters used in our experiments to ensure reproducibility. Unless otherwise specified, the same configuration is shared across all adaptation benchmarks. The EMA momentum μ is applied at every training iteration to update the teacher model. The DAM scaling factor ρ controls the relative contribution of the domain-aware matching cost and is kept fixed throughout training, while the annealing schedule is implicitly determined by the training progress. CAP-related parameters (β, κ, $\tau_{q}$, and *T*) are selected based on preliminary experiments and kept fixed across all benchmarks.

All experiments were conducted using PyTorch 2.8 with CUDA 12.8. The implementation is based on the Ultralytics RT-DETR codebase. Experiments were run on a single NVIDIA RTX 5090 GPU, under Ubuntu 22.04 and Python 3.12. All experiments in this paper are conducted with a fixed random seed (seed = 0). Due to computational constraints, results are reported from a single run. yield

### 4.3. Comparison with Other Methods

We compare SSRT-DETR with representative domain-adaptive detection baselines on three benchmarks. Table 2 reports results on Cityscapes→Foggy Cityscapes, Table 3 on KITTI→Cityscapes, and Table 4 on Sim10K→Cityscapes.

Table 2 reports adaptation performance on Cityscapes→Foggy Cityscapes. Compared with the RT-DETR-L source-only baseline, DAM* and CAP* each yields consistent gains, indicating that stabilizing Hungarian assignment and improving pseudo-label reliability are both beneficial under adverse weather shift. Notably, combining DAM + CAP* achieves the best overall mAP, suggesting that the two modules provide complementary improvements: DAM mainly reduces early-stage assignment noise, while CAP further suppresses pseudo-label errors under class imbalance and scene variability.

Table 5 presents a component-wise ablation study to analyze the contributions of the two key modules in SSRT-DETR. DAM and CAP are evaluated both individually and jointly on top of the same RT-DETR-L baseline.

When applied alone, DAM yields a consistent improvement over the baseline, indicating that stabilizing Hungarian matching is beneficial under domain shift. CAP delivers a greater performance gain when used independently, underscoring the importance of adaptive pseudo-label selection and weighting. Combining DAM and CAP yields further improvement, demonstrating that the two modules are complementary and address different failure modes in set-prediction, domain-adaptive detection.

Table 6 reports the per-class AP for two representative categories, *bicycle* and *train*, which are known to be sensitive to domain shift and class imbalance under foggy conditions.

For the *bicycle* class, introducing DAM improves AP over the baseline, indicating that stabilizing Hungarian matching is beneficial for objects with ambiguous boundaries. CAP alone yields a comparable but slightly lower AP, suggesting that adaptive pseudo-label filtering may be more conservative for this class. When DAM and CAP are combined, the bicycle AP recovers and reaches the highest level, showing that CAP benefits from the more stable assignments provided by DAM.

For the *train* class, which is relatively rare and prone to class confusion in Foggy Cityscapes, DAM alone leads to a noticeable AP drop. This behavior is expected, as stabilizing matching without explicitly controlling pseudo-label noise may still propagate semantically incorrect assignments for rare categories. In contrast, CAP significantly improves train AP by enforcing class-adaptive thresholds and semantic consistency. Combining DAM and CAP effectively compensates for the weaknesses of each component, resulting in a substantial recovery of train AP to near baseline levels while maintaining the overall performance gain. These observations further confirm the complementary nature of DAM and CAP.

As shown in Table 3, SSRT-DETR achieves the highest car AP on KITTI→Cityscapes. The strong improvement supports our motivation that DAM helps mitigate assignment errors in set-prediction detectors, thereby enabling more reliable self-training on the unlabeled target domain.

Table 4 presents results on synthetic-to-real adaptation (Sim10K→Cityscapes). The performance gain indicates that SSRT-DETR effectively handles large appearance discrepancies. CAP alleviates this issue by adapting confidence/IoU constraints and using quality-weighted supervision, leading to more stable target-domain learning.

Overall, SSRT-DETR consistently improves adaptation performance across adverse weather, cross-camera, and synthetic-to-real settings. These results validate the effectiveness of DAM in stabilizing Hungarian matching under domain shift and CAP in improving pseudo-label quality. Importantly, the proposed modules are training-time modifications and do not introduce additional inference-time overhead.

## 5. Discussion

This work targets a practical yet under-explored setting: adapting real-time set-prediction detectors to unlabeled target domains, where the one-to-one Hungarian assignment and pseudo-label self-training are both prone to error amplification under domain shift. Building upon RT-DETR, SSRT-DETR integrates a mean teacher–student paradigm with dual-view (real/fake) training, and further introduces two complementary mechanisms—Domain-Aware Matching (DAM) and Class-/Scene-Adaptive Pseudo-Labeling (CAP)—to enhance matching stability and pseudo-label reliability without altering the inference pipeline. It is worth noting that in single-class adaptation scenarios such as KITTI→Cityscapes, the class-adaptive components of CAP naturally degenerate to a class-agnostic form and therefore do not actively influence pseudo-label selection. In such cases, the observed performance gains primarily stem from the stabilization of Hungarian assignment provided by DAM, while CAP introduces no additional training or inference overhead.

### 5.1. Overall Effectiveness Across Adaptation Scenarios

The experimental results indicate that SSRT-DETR improves cross-domain detection performance on challenging scene shifts. On Cityscapes→FoggyCityscapes, SSRT-DETR yields consistent gains over representative DAOD baselines, with particularly notable improvements on fog-sensitive or visually ambiguous categories. For instance, large relative gains are observed for categories such as *bicycle* and *train*, which are typically vulnerable to contrast degradation and boundary ambiguity in adverse weather. On KITTI→Cityscapes (car-only adaptation), SSRT-DETR achieves a substantial improvement in target-domain AP compared with the source-only RT-DETR baseline, suggesting that the proposed training scheme effectively transfers localization and classification knowledge across camera/scene domains. These results collectively support the central claim that stabilizing assignment and calibrating pseudo-label selection are both critical for set-prediction DAOD.

### 5.2. Why DAM Matters for Set-Prediction Domain Adaptation

A key failure mode in DETR-like models under domain shift lies in early-stage assignment noise: when classification confidence and box regression are unreliable, the vanilla matching cost may select suboptimal query–object pairs, and the resulting supervision can further deteriorate training. DAM addresses this issue by augmenting the matching cost with a teacher–student query consistency term whose contribution is annealed over training progress. Conceptually, this domain-aware regularizer biases the matcher toward queries with more stable cross-view representations, helping mitigate spurious assignments in the early phase. Importantly, DAM operates inside the matching procedure and does not introduce additional inference-time components, making it compatible with real-time detectors.

### 5.3. Why CAP Improves Pseudo-Label Quality Under Class and Scene Variability

Pseudo-labeling with fixed thresholds is known to be brittle in cross-domain settings, especially when class frequencies are imbalanced, and scene conditions vary dramatically. CAP explicitly addresses this by (i) employing frequency-adaptive confidence thresholds, (ii) enforcing real–fake geometric consistency through class-adaptive IoU constraints, and (iii) incorporating prototype-based semantic verification to suppress class-confusion pseudo-labels that are geometrically stable yet semantically incorrect. Furthermore, the use of temperature scaling and quality-weighted distillation provides a soft mechanism to modulate the contribution of pseudo-label supervision, preventing low-quality pseudo-labels from dominating optimization. These design choices align with the empirical observation that the largest improvements tend to appear on categories and scenarios where domain shift most severely disrupts confidence calibration and semantic separability.

## 6. Conclusions

This paper presented SSRT-DETR, a semi-supervised domain-adaptive framework for real-time set-prediction detectors built upon RT-DETR. The proposed approach targets two key challenges in domain-adaptive detection: stabilizing Hungarian matching under domain shift and effectively exploiting noisy target-domain pseudo-labels without introducing additional inference cost.

To address the first challenge, Domain-Aware Matching (DAM) is formulated by augmenting the Hungarian matching cost with an annealed teacher–student query consistency term.

Experimental results across all adaptation scenarios demonstrate that DAM effectively stabilizes early-stage assignment and reduces error propagation, particularly under severe domain shift, while preserving the original inference pipeline.

To address the second challenge, we introduced Class-/Scene-Adaptive Pseudo-Labeling (CAP), which improves pseudo-label reliability through frequency-adaptive thresholds, multi-view geometric consistency, prototype-based semantic verification, and quality-weighted distillation. The effectiveness of CAP is validated in multi-class adaptation scenarios such as Cityscapes→FoggyCityscapes, where class imbalance and scene variability significantly affect pseudo-label quality. In single-class scenarios, CAP naturally degenerates to a class-agnostic form and does not alter training or inference behavior.

Overall, these results highlight the importance of jointly addressing assignment stability and pseudo-label quality control for domain adaptation in DETR-like detectors, and demonstrate that SSRT-DETR provides an effective and efficient solution for adapting real-time Transformer-based detection systems to unlabeled target domains.

## 7. Patents

The authors declare that no patents resulted from the work reported in this manuscript.

## Figures and Tables

**Table 1:** Training hyperparameters and configuration.

Parameter	Value
Optimizer	AdamW ($\beta_{1}=0.9,\beta_{2}=0.999$)
Base learning rate $lr_{0}$	$1\times 10^{-4}$
LR schedule	Warmup (10 epochs), cosine decay
Weight decay	$1\times 10^{-4}$
Batch size	2 (single GPU, no gradient accumulation)
Epochs	200
Input resolution	640×640 (resize, no crop)
Random seed	0
EMA momentum μ	0.9996
DAM scale ρ	0.5
CAP prototype update rate β	0.7
Sigmoid softness κ	0.5
Pseudo-label threshold $\tau_{q}$	0.7
Temperature *T*	2.0
Numerical stabilizer ϵ	$10^{-6}$

**Table 2:** Transfer the existing domain alignment method to RT-DETR and compare it with ours on Cityscapes→Foggy Cityscapes.

Method	Detector	Bus	Bicycle	Car	Mcycle	Person	Rider	Train	Truck	mAP (%)
Source Only	RT-DETR-L	46.7	55.0	75.6	38.0	58.4	55.8	**47.0**	31.7	51.0
DA-Faster [8]	Faster R-CNN	35.3	27.1	40.5	20.0	25.0	31.0	20.2	22.1	27.6
MAF [40]	Faster R-CNN	39.9	33.9	43.9	29.2	28.2	39.5	33.3	23.8	34.0
UMT [41]	Faster R-CNN	56.5	37.3	48.6	30.4	33.0	46.7	46.8	34.1	41.7
CMT [42]	Faster R-CNN	66.0	51.2	63.7	41.4	45.9	55.7	38.8	**39.6**	50.3
ConfMix [43]	YOLOv5	45.8	33.5	62.6	28.6	45.0	43.4	40.0	27.3	40.8
MTM [44]	DETR	28.8	28.0	68.8	23.8	53.7	35.1	41.6	37.2	48.9
DA-DETR [21]	DETR	45.9	46.5	64.1	32.6	49.9	50.0	33.8	25.8	43.5
DAM*	RT-DETR-L	64.5	**56.0**	78.1	38.2	58.9	56.3	39.5	27.4	52.3
CAP*	RT-DETR-L	**66.2**	54.5	**78.9**	41.5	59.1	**56.8**	43.6	31.5	54.0
DAM + CAP*	RT-DETR-L	62.2	**56.0**	78.8	**41.6**	**59.6**	**55.1**	46.2	35.0	**54.3**

**Note:** Bold values indicate the best performance in each column.

**Table 3:** Experimental results of the cross-camera adaptation: KITTI→Cityscapes.

Method	Detector	Car AP (%)
Source Only	RT-DETR-L	48.0
DA-Faster [8]	Faster R-CNN	38.5
CMT [42]	Faster R-CNN	64.3
ConfMix [43]	YOLOv5	52.2
DA-DETR [21]	DETR	48.9
RT-DATR [20]	RT-DETR	50.3
Our Method	RT-DETR-L	67.3

**Table 4:** Experimental results of the scenario synthetic scene to real scene: Sim10k→Cityscapes.

Method	Detector	Car AP (%)
Source Only	RT-DETR-L	49.9
DA-Faster [8]	Faster R-CNN	41.9
UMT [41]	Faster R-CNN	43.1
MTM [44]	DETR	58.1
ConfMix [43]	YOLOv5	56.3
DA-DETR [21]	DETR	54.7
Our Method	RT-DETR-L	64.9

**Table 5:** Component-wise ablation study on Cityscapes→Foggy Cityscapes. We analyze the individual and joint effects of Domain-Aware Matching (DAM) and Class-/Scene-Adaptive Pseudo-Labeling (CAP).

Setting	mAP@0.5 (%)	mAP@0.5:0.95 (%)
RT-DETR-L (baseline)	51.0	32.2
RT-DETR-L + DAM	52.3	33.5
RT-DETR-L + CAP	54.0	34.0
RT-DETR-L + DAM + CAP (SSRT-DETR)	54.3	34.8

**Table 6:** Component-wise ablation study on Cityscapes→Foggy Cityscapes. We analyze the individual and joint effects of Domain-Aware Matching (DAM) and Class-/Scene-Adaptive Pseudo-Labeling (CAP).

Setting	Bicycle AP	Train AP
RT-DETR-L (baseline)	55.0	47.0
RT-DETR-L + DAM	56.0	39.5
RT-DETR-L + CAP	54.5	43.6
RT-DETR-L + DAM + CAP (SSRT-DETR)	56.0	46.2

## Data Availability

All datasets are available on the official website.
